# Teletherapy Post‐COVID‐19: Comparisons With In‐Person Client Characteristics and Service Utilization in Routine Practice

**DOI:** 10.1002/jclp.70039

**Published:** 2025-08-26

**Authors:** Wilson T. Trusty, Brett E. Scofield, Stewart E. Cooper, Louis G. Castonguay, Jeffrey A. Hayes, Rebecca A. Janis

**Affiliations:** ^1^ Center for Counseling and Psychological Services Pennsylvania State University University Park Pennsylvania USA; ^2^ Psychological Sciences and Counseling Department Valparaiso University Valparaiso Indiana USA; ^3^ Department of Psychology Pennsylvania State University University Park Pennsylvania USA; ^4^ Department of Educational Psychology, Counseling, and Special Education Pennsylvania State University University Park Pennsylvania USA

**Keywords:** COVID‐19, psychotherapy, service utilization, telehealth, treatment outcomes

## Abstract

**Objective:**

Telehealth psychotherapy (TH) has become widespread since the COVID‐19 pandemic and has generally proven to be equally effective and acceptable as in‐person (IP) treatment. However, it is unclear how TH and IP client characteristics and service utilization compare in routine practice post‐pandemic.

**Methods:**

Individual psychotherapy clients (*N* = 22,710) receiving routine treatment at 72 university counseling centers from 2021 to 2023 reported on their demographic and clinical characteristics, while service utilization was determined through chart review.

**Results:**

Compared to IP clients, TH clients were more likely to identify as cisgender women, Hispanic/Latinx, and bisexual and were less likely to live with roommates. TH and IP clients reported roughly equivalent symptom severity at the beginning of treatment, but TH clients were less likely to report recent suicidal ideation and more likely to have a history of past psychotherapy and psychiatric medication use. Regarding service utilization, TH clients scheduled slightly more psychotherapy appointments yet attended a lower percentage of appointments, but these differences were small. Exploratory analyses on matched samples of TH and IP clients did not detect differences in pre‐ to posttreatment symptom reduction.

**Conclusion:**

In routine practice, TH and IP psychotherapy appear to facilitate access to care for slightly different client populations, are associated with similar utilization patterns, and produce similar outcomes in university counseling post‐COVID‐19. Clinical systems may best serve a diverse public by offering both TH and IP services. Policy‐level (e.g., equitable reimbursement for TH) and administrative support are needed to facilitate continued access to both modalities.

Providing psychotherapy via telehealth has become common since the COVID‐19 pandemic. A growing body of literature indicates that synchronous, video‐based telehealth (TH) and in‐person (IP) psychotherapy are equally effective for most clients (Bellanti et al. 2022; Davis et al. 2023; Gurm et al. 2023; Lalor et al. 2023; Thomas et al. 2021). However, research from early in the pandemic suggests that client characteristics and service utilization may differ between TH and IP in routine practice (e.g., a higher proportion of cisgender men using IP psychotherapy, higher attendance rates at TH appointments; Cuthbert et al. 2022; Neumann et al. 2023). This could affect access to care for certain clients depending on the availability of IP and TH treatment modalities. Differences in utilization between TH and IP psychotherapy could also impact clinical systems, such as if clients in a particular modality tend to consume more services. Because TH care continues to shift in the postpandemic era (Calkins 2022), an updated knowledge of these potential differences in clients and utilization between TH and IP psychotherapy in routine practice could inform efforts to both provide equitable access to care and to allocate resources effectively. Despite this, little data since the early pandemic is available to determine if client characteristics or service utilization currently differ between TH and IP treatment. To address this gap, the present study compared client demographic and clinical characteristics and service utilization in IP and TH psychotherapy in a national United States sample of university counseling center psychotherapy clients from 2021 to 2023.

## 1 Comparisons of Telehealth and In‐Person Psychotherapy

Much of the research comparing client characteristics and utilization early in the COVID‐19 pandemic was based on the hypothesis that TH would increase access to care among previously underserved populations (Connolly et al. 2021). For example, TH could reduce the stigma of attending psychotherapy for certain groups or be more easily accessible to individuals without reliable transportation (Bailey et al. 2021; Truong et al. 2022). This could lead to differences in demographic or clinical characteristics of clients TH and IP psychotherapy, such as a higher proportion of individuals with marginalized identities or more functional impairment receiving TH services and attending sessions more regularly.

The empirical evidence on client demographic characteristics has provided mixed support for the idea that TH would facilitate access to care for more diverse groups (Mahtta et al. 2021). For example, a review of TH services for sexual and gender minority (SGM) clients that spanned 2018‐2022 suggested that TH expansion during the pandemic did lessen the impact of stigma on help‐seeking for this population (Whaibeh et al. 2022). However, it was unclear whether the proportion of SGM clients in TH was actually higher than IP. A systematic review of studies published from 2020 to 2022 found that women were more likely to receive treatment via TH than IP psychotherapy, but there were no consistent differences for other demographic characteristics such as race/ethnicity or living situation (Neumann et al. 2023). Other studies using data from 2021 to 2022 have similarly found limited evidence for demographic differences between TH and IP clients (Connolly et al. 2021; Tobin et al. 2023; Vakil et al. 2022), potentially because disparities in access to care that were present in IP care persisted in TH (e.g., those lacking transportation also being less likely to have reliable internet; Mahtta et al. 2021).

Considering clinical characteristics, although presenting concerns and diagnosis were unrelated to the use of TH treatment in 2020 (Miu et al. 2020; Severe et al. 2020), clients with higher symptom severity at intake were more likely to be seen via IP (Neumann et al. 2023). In line with this, a large study of over 1,000,000 individuals receiving psychotherapy or psychiatry in 2020 in the Department of Veterans Affairs found that those who attended the majority of appointments via TH were less likely to have schizophrenia spectrum disorders or a history of psychiatric hospitalization (Connolly et al. 2021). Although this contrasts with expectations that TH may facilitate access to care for more severely impaired clients, it is consistent with clinician concerns about caring for clients with elevated risk via TH, such as those with suicidal ideation (Gilmore and Ward‐Ciesielski 2019). Despite higher symptom severity in IP care, rates of risk of harm to others (e.g., engaging in physical fights or carrying weapons) were higher among youth receiving TH versus IP treatment in 2021 (Emezue et al. 2024).

In terms of service utilization, research has reported small but consistent differences between IP and TH in the early stages of the pandemic. In a naturalistic IP‐TH comparison (Gurm et al. 2023), 2788 clients who received TH psychotherapy at a community mental health center between March 2020 and March 2021 attended slightly more sessions (*M* = 5.14) than a matched sample who received IP services in the 5 years before the pandemic (*M *= 4.48 sessions). Related, naturalistic data from a cognitive behavioral therapy specialty clinic indicated that no‐show rates were lower in TH (9.94%) than IP psychotherapy (12.84%) during a 6‐year period that included the beginning of the pandemic among 1869 clients (Cuthbert et al. 2022). Similar findings regarding higher attendance rates in TH have been reported in other outpatient (Connolly Gibbons et al. 2024), intensive outpatient (Childs et al. 2021), and psychiatric settings (Muppavarapu et al. 2022) in the first year of COVID‐19. Taken together, findings are generally consistent with the expectation that TH would facilitate more regular attendance.

While these results are informative, the majority are based on data from 2020 to 2021 (e.g., Connolly Gibbons et al. 2024; Connolly et al. 2021; Emezue et al. 2024; Tobin et al. 2023; Vakil et al. 2022). TH care has undergone significant changes since this period, though. For instance, client characteristics and treatment utilization could have shifted since 2021 due to wider availability of both IP and TH psychotherapy services with the easing of COVID‐19 risk mitigation policies (Calkins 2022), increased familiarity with telehealth among clients and therapists, or efforts to address inequities in access to TH (Health Resources and Services Administration 2024). Additionally, certain differences in client characteristics in routine TH psychotherapy remain largely untested. For instance, even though clinicians have raised concerns about treatment of clients with suicidal ideation via TH (Gilmore and Ward‐Ciesielski 2019), it is unclear whether suicidality is less prevalent among TH clients. As such, it is unknown whether past findings comparing client characteristics and utilization reflect current differences between TH and IP clients. Further, the majority of research examining differences between TH and IP psychotherapy has been conducted in community mental health and military settings (e.g., Gurm et al. [2023]; Neumann et al. [2023]). An updated analysis in a wider variety of settings could clarify who is accessing IP and TH psychotherapy and how those individuals use services. This information might elucidate the types of interventions that clients in both modalities may prefer or need as well as the impact that these services have on clinical systems.

## 2 The Present Study

The present study compared client characteristics and service utilization in routine practice at university counseling centers post‐COVID‐19 (i.e., from 2021 to 2023). Since no prior research has compared these variables in TH and IP psychotherapy at university counseling centers, we aimed to test whether differences found in other outpatient settings early in COVID‐19 replicated in routine, postpandemic university counseling. First, we explored whether clients in TH and IP psychotherapy differed in their demographic characteristics, specifically gender, sexual orientation, race/ethnicity, and living situation. We selected these variables to facilitate comparison with research on demographic differences between TH and IP clients early in the pandemic (Neumann et al. 2023; Whaibeh et al. 2022). Examination of these variables could also be useful for clinical systems, as they are often assessed in routine practice. Second, we compared clients' clinical characteristics between modalities. Based on research showing higher symptom severity in IP treatment (Neumann et al. 2023), clinician‐reported concerns regarding TH treatment of clients with suicidality (Gilmore and Ward‐Ciesielski 2019), and potential differences in risk of harm to others between IP and TH (Emezue et al. 2024), we hypothesized that those treated in person would report greater symptom severity and risk‐related variables (i.e., suicidal and homicidal ideation). We also explored differences in treatment history between IP and TH clients since this can be related to risk level (e.g., history of psychiatric hospitalization). Third, in line with prior findings on service utilization (Cuthbert et al. 2022; Gurm et al. 2023), we hypothesized that TH clients would schedule more psychotherapy appointments and attend a greater proportion of appointments than IP clients. Finally, we conducted exploratory analyses testing whether TH and IP clients differed in treatment outcomes after accounting for potential discrepancies in demographic and clinical characteristics.

## 3 Methods

### 3.1 Participants

Participants were 22,710 individual psychotherapy clients at 72 university counseling centers (UCCs) in the United States that offered both TH and IP services between July 2021 and December 2023. Centers were members of the Center for Collegiate Mental Health (CCMH), which is a practice‐research network of UCCs (Locke, Bieschke et al. 2012). To be included, clients had to have attended at least two individual psychotherapy sessions and have demographic and clinical characteristics on file. They also had to have completed at least two administrations of the Counseling Center Assessment of Psychological Symptoms (CCAPS; Locke, McAleavey et al. 2012): one within 14 days of their first attended appointment and another within 14 days of their last. To avoid the confound of participants receiving multiple modes of treatment, clients had to have all their scheduled appointments be either IP or video‐based TH. Sample demographic and clinical characteristics are shown in Tables 1 and 2, respectively.

**Table 1:** Sample demographic characteristics.

Variable	Full sample % (*N* = 22,710)	In‐person % (*n* = 15,434)	Telehealth % (*n* = 7,276)	Odds ratio	*p* value
Gender					
Cisgender man	28.7	31.8	22.2	0.63	< 0.001*
Cisgender woman	58.1	55.9	62.9	1.52	< 0.001*
Nonbinary	3.1	3.1	3.0	1.02	0.835
Transgender man	0.7	0.6	0.7	1.19	0.329
Transgender woman	0.4	0.4	0.4	1.04	0.911
Self‐Identify	1.1	1.1	1.1	1.00	0.999
Race/Ethnicity					
African American/Black	8.6	8.4	9.1	1.08	0.117
American Indian or Alaska Native	0.4	0.4	0.3	0.73	0.213
Asian American/Asian	9.3	9.8	8.2	0.82	< 0.001*
Hispanic/Latinx	12.1	11.4	13.6	1.22	< 0.001*
Native Hawaiian or Pacific Islander	0.2	0.2	0.2	1.33	0.419
Multi‐Racial	5.2	5.0	5.7	1.16	0.020
White	59.6	60.1	58.6	0.92	0.003
Self‐identify	1.4	1.3	1.5	1.12	0.362
Sexual orientation					
Asexual	2.4	2.4	2.3	0.92	0.429
Bisexual	13.4	12.8	14.7	1.15	0.001*
Gay	2.3	2.2	2.4	1.03	0.739
Heterosexual	57.9	58.3	57.1	0.89	< 0.001*
Lesbian	2.4	2.2	2.7	1.22	0.028
Pansexual	3.0	2.9	3.2	1.11	0.225
Queer	2.8	2.7	3.0	1.10	0.264
Questioning	3.6	3.6	3.6	0.98	0.791
Self‐identify	1.0	1.1	1.0	0.90	0.484
Living situation					
Alone	11.2	10.2	13.4	1.35	< 0.001*
Significant other	7.6	6.3	10.3	1.71	< 0.001*
Roommates	58.7	62.3	51.1	0.63	< 0.001*
Children	1.4	1.0	2.2	2.19	< 0.001*
Parents	9.1	8.2	11.2	1.42	< 0.001*
Other family	4.4	4.0	5.1	1.28	< 0.001*
Other	1.0	1.0	1.2	1.30	0.050
Financial stress (*M* [SD])	3.0 (1.1)	3.1 (1.1)	3.0 (1.1)	—	—
Age (*M* [SD])	21.7 (4.1)	21.3 (3.8)	22.4 (4.7)	—	—

*Note: p* values are for Fisher's exact test. Financial stress scores range from 1 to 4, with higher scores indicating higher stress about one's financial situation.

* *p* value is significant after applying the Holm–Bonferroni correction.

**Table 2:** Sample clinical characteristics.

Variable	Full sample % (*N* = 22,710)	In‐person % (*n* = 15,434)	Telehealth % (*n* = 7276)	Odds ratio	*p* value
Treatment history					
Prior psychotherapy	46.8	44.0	52.8	1.28	< 0.001*
Prior psychiatric medication	27.7	25.7	32.0	1.29	< 0.001*
Prior psychiatric hospitalization	7.0	6.6	7.7	1.13	0.024
Risk to self and others					
Suicidal ideation (lifetime)	30.5	30.7	30.1	0.98	0.538
Suicidal ideation (past 2 weeks)	3.3	3.6	2.7	0.74	< 0.001*
Homicidal ideation (lifetime)	5.1	5.2	4.7	0.89	0.069
Homicidal ideation (past 2 weeks)	0.7	0.7	0.6	0.87	0.511
Primary presenting concern					
Generalized anxiety	11.8	11.2	13.1	—	—
Depression	10.8	10.8	10.8	—	—
Relationship problem	4.5	4.4	4.8	—	—
Stress	3.9	3.7	4.5	—	—
Trauma	3.4	3.3	3.6	—	—
Social anxiety	3.3	3.4	3.2	—	—
Family	3.1	3.1	3.1	—	—
Adjustment to new environment	2.7	2.7	2.6	—	—
Interpersonal functioning	2.3	2.4	2.2	—	—
Grief/loss	2.3	2.1	2.8	—	—
Unspecified/other anxiety	1.7	1.5	2.2	—	—
Academic performance	1.6	1.7	1.5	—	—
Self‐esteem/confidence	1.5	1.7	1.2	—	—
Eating/body image	1.3	1.4	1.3	—	—
Emotion dysregulation	1.3	1.2	1.3	—	—
Panic attacks	1.3	1.1	1.7	—	—
Attention/concentration difficulties	1.2	1.2	1.1	—	—
Sexual abuse/assault	1.1	1.0	1.3	—	—
Suicidality	1.0	1.2	0.7	—	—

*Note: p* values are for Fisher's exact test. The 20 most frequent presenting concerns are shown.

* *p* value is significant after applying the Holm–Bonferroni correction.

### 3.2 Measures

#### 3.2.1 Standardized Data Set Client Information Form

The Client Information Form is a component of CCMH's Standardized Data Set (SDS; Center for Collegiate Mental Health 2022), which gathers demographic information and clinical characteristics (e.g., treatment history). It also asks clients to indicate whether and when they have “seriously considered attempting suicide” or “considered causing serious physical injury to another person.” We operationalized the presence of suicidal and homicidal ideation in two ways: (1) lifetime history, defined as clients endorsing ever experiencing either of these symptoms, and (2) recent ideation, defined as clients reporting these symptoms were present in the past 2 weeks.

#### 3.2.2 Counseling Center Assessment of Psychological Symptoms

The Counseling Center Assessment of Psychological Symptoms (CCAPS; Locke, McAleavey et al. 2012) is a self‐report routine outcome monitoring instrument commonly used in UCCs. The 34‐item version (CCAPS‐34) was used in the present study. The CCAPS‐34 contains the following subscales: Depression, Generalized Anxiety, Social Anxiety, Academic Distress, Eating Concerns, Frustration/Anger, and Alcohol Use. It also produces a measure of general distress (the Distress Index), which is calculated using key items from the subscales. Participants rate symptoms on a scale of 0 (*not at all like me*) to 4 (*extremely like me*) based upon the past 2 weeks. The Distress Index and mean of each subscale were used as the indicators of symptom severity. The CCAPS‐34 subscales have shown good convergent and discriminant validity and internal consistency (Locke, McAleavey et al. 2012; McAleavey et al. 2012; Youn et al. 2015) and have demonstrated good 2‐week test–retest reliability (Locke, McAleavey et al. 2012) and sensitivity to change (Youn et al. 2020).

#### 3.2.3 Attendance and Service Utilization

Client attendance and utilization information was gathered through reviewing records of appointment data. Utilization was calculated as the sum of psychotherapy appointments a client scheduled regardless of whether they attended, while attendance rates were the percentage of scheduled appointments a client attended.

### 3.3 Procedure

Clients completed the Client Information Form and CCAPS‐34 at the beginning of treatment; they completed the CCAPS‐34 again within 14 days of the end of treatment. Assignment to TH or IP psychotherapy and the number of sessions scheduled were determined locally by clients and providers according to their standard practices. Participating centers sent deidentified client data to CCMH's central repository for research purposes. All study procedures were approved by the IRB at the first author's institution and by the institutions of all UCCs involved before data collection.

### 3.4 Data Analysis

To test differences in demographic and clinical characteristics between IP and TH clients, we created dichotomous variables for each characteristic and conducted separate *χ* ^2^ analyses comparing prevalence between TH and IP treatment. We used the sequential Holm–Bonferroni correction to hold the family‐wise error rate at 0.05 (Abdi 2010; Eichstaedt et al. 2013). To test the hypothesis that clients in IP psychotherapy would report more severe symptoms, we conducted a multivariate analysis of variance (MANOVA) with all CCAPS‐34 subscales and the Distress Index as dependent variables and mode of treatment (TH vs. IP) as the independent variable. If the omnibus test was significant, we planned to use post hoc *t*‐tests to examine differences for individual CCAPS scales. All subscales were approximately normally distributed except for Eating Concerns and Alcohol Use, and so we used log transformed versions of these two variables.

To compare attendance between TH and IP psychotherapy, we conducted a Mann–Whitney *U* test with percentage of scheduled appointments attended as the dependent variable and treatment modality as the independent variable. We used a *U* test because the attendance variable was highly non‐normal and was not improved with transformations. To examine service utilization, we conducted an independent samples *t*‐test with treatment modality as the independent variable and number of sessions scheduled as the dependent variable. Because number of sessions scheduled was highly skewed to fewer sessions, we used a log transformed version of the variable.

Finally, we explored potential differences in outcomes on the CCAPS between TH and IP. As has been done in previously (Davis et al. 2023; Gurm et al. 2023), we constructed matched groups using the nearest neighbor method without replacement with the MatchIt package in R 4.2.2 (Ho et al. 2007). We opted to use nearest neighbor matching because it (1) mitigates problems with multicollinearity that can arise with large numbers of covariates, (2) typically maintains statistical power to identify differences in outcomes even with a reduced sample size due to smaller standard deviations, and (3) is robust to potential lack of overlap in covariates between treatment groups (Stuart 2010). We created the matched samples based on characteristics hypothesized to differ between groups based on past literature: gender identity, race/ethnicity, sexual orientation, treatment history, suicidal ideation in the 2 weeks before intake, living situation, and baseline CCAPS Distress Index score. We calculated symptom change scores for each CCAPS scale by subtracting clients' final score from their first. We planned to conduct a MANCOVA with change scores as dependent variables, mode of treatment as the independent variable, and any variables that differed between groups after matching as covariates. We only controlled for variables that differed between groups because the goal of this exploratory analysis was to test whether clients in TH versus IP experienced different outcomes given similar clinical and demographic characteristics (i.e., we did not aim to test whether modality predicted outcomes above and beyond correlates of symptom change, which could make it more difficult to detect small but potentially meaningful outcome differences between groups). In this context, controlling for variables that did not differ between groups would be redundant. For example, if age or baseline distress were equal between matched samples, then differences in these variables would be automatically ruled out as alternative explanations for any between‐group outcome differences. If the MANCOVA predicting change scores was significant, we planned to follow up with post hoc *t*‐tests. Because the MatchIt package requires all data to be present, clients with missing data on any of the indicators above were excluded. We did not impute missing data because many of the characteristics (e.g., living situation) would not be expected to be predicted accurately from the other variables.

## 4 Results

First, we explored whether TH and IP clients differed in demographic characteristics (e.g., gender identity, race/ethnicity, and sexual orientation). Descriptive and inferential statistics are shown in Table 1. TH clients were less likely to identify as cisgender men and more likely to identify as cisgender women. A greater proportion of TH clients identified as Hispanic/Latinx, while fewer identified as Asian/Asian American. Additionally, TH clients were more likely to identify as bisexual and less likely to identify as heterosexual, and they were less likely to live with roommates and more likely to report other living arrangements (e.g., alone, with a significant other). This indicates that TH and IP clients differed on multiple demographic variables.

Second, we tested the hypothesis that IP clients would report higher symptom severity and risk‐related variables than TH clients; we also explored differences in treatment history between groups. The MANOVA comparing baseline symptom severity between TH and IP clients was significant, *F*(1, 8) = 6.54, *p* < 0.001, indicating that baseline symptoms differed between groups. Post‐hoc analyses showed that TH clients reported slightly lower scores on the Distress Index, Frustration/Anger, and Alcohol Use scales and slightly higher scores on Generalized Anxiety and Eating Concerns (see Table 3). However, all effects were extremely small (*d*s ranging from 0.01 to 0.04). Descriptive and inferential statistics for risk variables and treatment history are shown in Table 2. IP clients were more likely to report suicidal ideation in the 2 weeks before intake, but no significant differences in other risk variables were observed. TH clients were more likely to report prior psychotherapy and psychiatric medication than IP clients, though there were no differences in hospitalization history. Taken together, this suggests that there were no meaningful differences in symptom severity between TH and IP clients, but recent suicidal ideation was more common in IP psychotherapy and outpatient mental healthcare histories were more prevalent among TH clients.

**Table 3:** Baseline CCAPS‐34 scores.

CCAPS‐34 scale	Full sample (*N* = 22,710)	In‐person (*n* = 15,434)	Telehealth (*n* = 7276)	*t*	*p* value	*d*
Depression	1.66	1.67	1.64	1.84	0.066	0.03
Generalized anxiety	2.03	2.02	2.05	−2.24	0.025	0.03
Social anxiety	2.13	2.13	2.11	1.41	0.158	0.02
Academic distress	1.96	1.96	1.96	−0.57	0.572	0.01
Eating concerns	1.03	1.02	1.06	−2.30	0.021	0.03
Frustration/anger	0.78	0.79	0.76	2.89	0.004	0.04
Alcohol use	0.44	0.45	0.43	2.10	0.036	0.03
Distress Index	1.79	1.79	1.78	0.67	0.500	0.01

*Note:* Inferential statistics for eating concerns and alcohol use are based on log‐transformed versions of these variables.

Third, we tested the hypothesis that service utilization and attendance would be higher among TH clients. The number of appointments scheduled in TH (*M* = 6.50, SD = 4.76) was slightly higher than IP (*M* = 6.06, SD = 4.09), *t*(13,784) = 6.07, *p* < 0.001, *d* = 0.09. In contrast, the percentage of IP appointments attended (median = 0.86) was slightly higher than TH (median = 0.83), *z* = 5.85, *p* < 0.001, *r* (rank biserial) = 0.05. This indicates that utilization and attendance were nearly equal between groups, but TH clients scheduled marginally more sessions and attended a slightly lower percentage of those sessions than IP clients.

Finally, we explored differences in treatment outcomes between TH and IP psychotherapy clients. Propensity score matching resulted in samples of 1547 TH and 1547 IP clients. Comparisons of IP and TH client characteristics and diagnostic indices for the matching procedure are in the Supporting Information. Diagnostic indices indicated good matching between groups (i.e., standardized mean differences for all variables were below 0.1; Stuart et al. 2013). The only characteristics that significantly different between groups were the percentages of cisgender men and women and baseline scores on the Eating Concerns CCAPS subscale. As such, we added gender and Eating Concerns as covariates when examining differences in outcomes. Client age did not differ between groups (see Supporting Information) and did not predict pre‐ to posttreatment change in the Distress Index, *r*(3,092) = 0.01, *p* = 0.668), and so age was not included as a covariate in the analyses. Because number of sessions attended has been shown to relate to symptom change in UCC psychotherapy (Erekson et al. 2015; Trusty, Castonguay et al. 2024), we tested whether this variable differed between groups. Telehealth clients attended 0.54 more sessions than IP clients on average (*M* = 5.54, SD = 4.29, vs*. M* = 5.10, SD = 3.82), *t*(3,081) = 2.89, *p* = 0.004, *d* = 0.10. Thus, we added number of attended sessions as a covariate in outcome comparisons. MANCOVA results indicated that there were no significant differences in symptom change on the CCAPS subscales between modalities after controlling for gender, Eating Concerns, and number of attended sessions, *F*(1, 8) = 1.16, *p* = 0.319. Paired samples *t*‐tests comparing first and last CCAPS administrations within the IP and TH groups showed significant improvement on all CCAPS subscales. Change scores and effect sizes are displayed in Table 4.

**Table 4:** Within‐group symptom change between first and last CCAPS‐34 administrations.

CCAPS‐34 scale	In‐person (*n* = 1547)			Telehealth (*n* = 1547)		
	*M*(SD)	*d*	*p* value	*M*(SD)	*d*	*p* value
Depression	0.60(0.90)	0.60	< 0.001	0.62(0.89)	0.63	< 0.001
Generalized anxiety	0.48(0.79)	0.50	< 0.001	0.50(0.81)	0.51	< 0.001
Social anxiety	0.33(0.69)	0.35	< 0.001	0.33(0.68)	0.35	< 0.001
Academic distress	0.26(0.92)	0.26	< 0.001	0.27(0.92)	0.26	< 0.001
Eating concerns	0.30(0.86)	0.26	< 0.001	0.35(0.93)	0.28	< 0.001
Frustration/anger	0.35(0.64)	0.46	< 0.001	0.37(0.64)	0.50	< 0.001
Alcohol use	0.15(0.58)	0.22	< 0.001	0.21(0.61)	0.28	< 0.001
Distress Index	0.49(0.65)	0.64	< 0.001	0.51(0.67)	0.65	< 0.001

*Note:* Positive scores indicate reduction in symptoms (i.e., symptom improvement). Results were calculated from the matched sample of in‐person and telehealth clients.

## 5 Discussion

The present study compared client characteristics and service utilization in TH and IP psychotherapy in routine university counseling center practice post‐COVID‐19. First, we found a number of differences in demographic characteristics between IP and TH clients. For instance, TH clients had a 52% higher odds of identifying as cisgender women, 22% higher odds of identifying as Hispanic/Latinx, 11% lower odds of identifying as heterosexual, and 37% lower odds of living with roommates. There were no meaningful differences in symptom severity between modalities, but TH clients were more likely to report prior psychotherapy and psychiatric medication and less likely to report recent suicidal ideation. Related to service utilization, IP clients had slightly higher attendance and scheduled somewhat fewer total appointments than those in TH, but the sizes of these effects were small. To illustrate the size of utilization effects, if a hypothetical UCC with 20 clinical staff members served 1,000 clients a year and treated one‐third of these via TH, each clinician would need to schedule approximately seven additional TH sessions per year to accommodate the slightly higher number of sessions that TH clients schedule; despite this, attendance at these sessions may be slightly lower than at IP sessions. Finally, exploratory analyses detected no differences in symptom reduction between matched samples of TH and IP clients. While it remains to be tested whether these findings apply to other treatment settings (e.g., community mental health centers, private practice), this pattern of results suggests equivalent treatment outcomes but some differences in who accesses TH and IP psychotherapy and how these services are utilized in university counseling centers.

These results have both consistencies and differences compared to research on TH and IP psychotherapy near the beginning of COVID‐19. Similar to previous findings (Neumann et al. 2023), cisgender women were more likely to use TH in the present study. However, TH clients in the present study were slightly more diverse in terms of sexual orientation and more likely to identify as Hispanic/Latinx. This suggests that telehealth psychotherapy may facilitate treatment access among certain marginalized groups in the current cultural context, though IP services may increase access to care for other groups (e.g., Asian/Asian American clients or college students living with roommates, who were more likely to be treated in person).

While IP clients reported more severe symptoms before and during the early pandemic (Neumann et al. 2023), they did not differ substantially in their symptoms in the present study. In fact, those in TH were more likely to report a history of prior psychotherapy and psychiatric medication, which could indicate more chronic concerns in the TH group. The exception, however, was recent suicidal ideation, which was more common among IP clients. This is consistent with prior research showing that TH clients in routine practice were less likely to have a history of psychiatric hospitalization (Connolly et al. 2021). However, clinical trials of suicide‐specific interventions indicate that TH is effective for treating suicidality (Shoib et al. 2024). It may be that provider concerns about managing suicidal risk via TH result in more frequent assignment of clients with recent suicidal ideation to IP (Gilmore and Ward‐Ciesielski 2019). Alternatively, third variables that could affect both suicidal ideation and assignment to IP psychotherapy may be responsible (e.g., financial difficulties contributing to suicidal ideation and precluding having the technology or private space to join TH sessions).

Finally, the differences in attendance and appointments scheduled between TH and IP were smaller in the current analyses than near the beginning of the pandemic (Childs et al. 2021; Cuthbert et al. 2022; Gurm et al. 2023; Muppavarapu et al. 2022). This could be an artifact of the present study using a university counseling center sample (e.g., close proximity to an on‐campus counseling center may diminish differences in attendance between modalities among college students). Alternatively, this could indicate that clients are utilizing TH and IP appointments similarly as TH services have become more mainstream. Indeed, while our data do not examine changes over time, comparisons of research from early in the pandemic with the pattern of results in the present study suggests that TH psychotherapy may be becoming more utilized by diverse clients with more comparable levels of symptoms to those in IP (perhaps due to greater clinician experience and comfort with TH) and that differences in how clients engage with TH and IP treatment may be lessening. Importantly, outcomes still appear to be similar between modalities despite these potential shifts.

### 5.1 Limitations and Future Directions

While the present study has several strengths (e.g., large naturalistic data set; use of a well validated outcome monitoring instrument for the population studied; objective measurement of treatment utilization variables), it also has limitations. Notably, the sample consisted solely of UCC clients. Thus, some of the differences in findings between the present study and past research could be due to the use of different populations. Related, the present study only examined data from 2021 to 2023 rather than investigating changes in client characteristics and utilization since the beginning of the pandemic. This precludes making direct comparisons with data from the early pandemic within the population studied. Additionally, the present study did not examine center‐level characteristics that could account for differences between TH and IP treatment. For instance, even though all centers offered both IP and TH, local policies and culture regarding TH could result in differences between this study's results and the clinical makeup of TH and IP clients at individual centers. To address these limitations, research is needed to replicate these findings in other settings (e.g., community mental health centers), examine longitudinal changes in client characteristics and utilization in TH and IP psychotherapy over time, and investigate center‐level predictors of differences in client characteristics and utilization between IP and TH treatment.

In addition to addressing limitations of the present study, further research is needed to understand why certain populations are more likely to receive TH or IP psychotherapy across centers. For example, there was a slightly higher proportion of clients of each sexual orientation besides heterosexual in TH than IP (though the difference was only statistically significant for bisexual and heterosexual clients). Past writing has suggested that this could be due to reduced stigma, increased access to culturally competent providers, or easier access to services for LGBTQ+ clients via telehealth (Whaibeh et al. 2020, 2022). Future research could elicit feedback from LGBTQ+ individuals on their perceptions on TH versus IP psychotherapy to further clarify what factors impact their preferences for a certain treatment modality and learn which are applicable in other settings besides university counseling centers. Additionally, research on potential preferences for one modality over another should be extended to other treatment settings besides counseling centers. These results could help inform local clinical practices. As one example, if LGBTQ+ clients report that concerns about discrimination in a mental health clinic is a barrier to seeking IP care, clinics could evaluate the extent to which their advertising, online materials, and physical spaces are welcoming to sexually and gender‐diverse clients; identify strategies to increase inclusiveness (e.g., through waiting room signs or brochures that are LGBTQ+‐affirming); and monitor LGBTQ+ clients' use of and comfort with TH and IP modalities.

Examining the reasons why other groups of clients more frequently receive TH or IP psychotherapy would also be valuable. For instance, future research could test multiple potential explanations for the small racial/ethnic differences in use of IP and TH seen in the present study, such as differences in access to transportation to IP appointments, high‐speed internet, or private space to join TH appointments; culturally bound client preferences for one modality over another; stigma for attending psychotherapy; or clinicians' judgments about the use of TH and IP modalities with certain types of clients. This could inform advocacy efforts (e.g., supporting measures to secure equitable access to high‐speed internet) and clinical practices (e.g., identifying and addressing biases about clients with certain racial/ethnic identities being appropriate for IP or TH care). Additionally, future research could clarify whether associations of certain variables with treatment modality are an artifact of other client characteristics (e.g., whether the higher prevalence of prior psychotherapy among TH clients is due to more White, cisgender women in TH, who are also more likely to have had psychotherapy in the past; Magaard et al. [2017]). A better understanding of these processes could inform efforts to address potential disparities in access to TH and IP care.

While differences in TH and IP clients' number of scheduled and attended sessions were quite small, additional research on whether these differences are consistently present and why they exist could be valuable. The fact that TH clients scheduled slightly more sessions could be due to a variety of reasons, such as the higher proportion of clients with a history of mental healthcare receiving TH (i.e., those with more chronic concerns requiring slightly more sessions to reach a positive outcome). Alternatively, it could be that the rate of symptom change is slightly slower in TH regardless of client characteristics. Clarifying the reasons for discrepancies in appointments could help clinical systems understand how offering TH and IP may affect their clinical capacity over time. Similarly, while differences in attendance between IP and TH were minor, understanding the reasons for this could help improve attendance in TH, particularly in university counseling settings (e.g., reducing distractions in the home that could interfere with attendance, improving internet access).

Finally, additional research is needed to guide assignment of clients to various treatment modalities in routine practice. Many decades ago, Gordon Paul proposed his “matching hypothesis” (Paul 1967) that the effectiveness of psychotherapy depends on fit between (1) the client's needs and characteristics, (2) the therapist's approach and style, and (3) the specific treatment or intervention used. Several classic articles and more recent meta‐analyses provided support for the matching hypotheses (Calvert et al. 1988; Luborsky et al. 1988; Norcross 2011; Smith et al. 1980). A fourth addition now needs to be studied: the modality of service delivery, including TH or IP. Future research should focus on the simultaneous evaluation of all four of these dimensions throughout clients' courses of treatment. This study may consider not only treatment outcomes, but also potential differences in psychotherapy process variables associated with outcomes in (e.g., the working alliance; Davis et al. 2023).

The current study has added to the literature suggesting that modality of service delivery may matter more to access than to outcomes. However, there may also be differences in outcomes for clients with certain combinations of characteristics (e.g., combinations of identities, treatment histories, and symptom severity; Trusty, Scofield et al. 2024). Consistent with the principles of routine outcome monitoring (Barkham et al. 2023), clinicians and clients may also determine that the treatment modality needs to switch to improve outcomes during a single course of psychotherapy (e.g., if a client beginning psychotherapy with low symptom severity deteriorates in TH, they may need to switch to IP care to ensure safety and positive outcomes). Future research could test this by employing sequential RCT designs (e.g., Guidi and Fava 2021). For example, those who do not show reliable symptom improvement in a randomly assigned modality (TH vs*.* IP) could then be randomized to either continue with their current modality—perhaps with a different therapist or approach—or switch to the other modality to test whether this improves outcomes. Such designs could help inform whether changes in modality are indicated for certain clients during treatment.

## 6 Conclusions

The present study suggests that TH and IP psychotherapy facilitate access to care for slightly different populations, are associated with similar utilization and attendance rates, and have comparable outcomes post‐COVID‐19. Consistent with past research, we found nuanced differences in the types of clients that use TH versus IP psychotherapy since the COVID‐19 pandemic. Thus, while TH psychotherapy may increase access to care for certain groups (e.g., Hispanic/Latinx or bisexual clients), IP services appear to be somewhat more commonly used by other populations (e.g., Asian American/Asian clients or those who may not have private space to join TH appointments). Because of this, clinical systems may best serve a diverse clientele by offering both TH and IP modalities. Policymakers and administrators can facilitate this by supporting measures to protect provision of TH and IP care, such as advocating for equitable reimbursement for TH services (American Psychological Association nd) and retaining access to IP services on college campuses and other settings. Notably, the present findings suggest that individual clinicians and clinical systems may offer both modalities without significant impacts on their clinical capacity or treatment outcomes.

The present study also indicates that a substantial proportion of TH clients are currently entering treatment with a history of prior outpatient mental healthcare. As such, clinicians seeing TH clients may more frequently need to obtain information from prior providers to fully assess clients’ needs and make appropriate referrals (e.g., to begin concurrent medication with a psychiatric prescriber). Despite this, clients with recent suicidal ideation appear to be treated less frequently in TH. Given the evidence that TH suicide‐focused interventions are highly effective (Shoib et al. 2024) and the present findings that clients of certain marginalized backgrounds are slightly more likely to receive TH care, creating flexibility for clients with suicidal risk to receive TH psychotherapy might increase access to care for these individuals. Clinicians and clinic leadership may help reduce potential inequities by critically examining practices regarding assignment to TH versus IP psychotherapy for clients with suicidal risk, obtaining necessary training in TH provision of suicide‐focused treatment, and monitoring the ongoing appropriateness of a given mode of treatment for clients on a case‐by‐case basis.

## Conflicts of Interest

The authors declare no conflicts of interest.

## Supporting information


**Supporting Table S1:** Demographic characteristics of the matched samples. **Supporting Table S2:** Clinical characteristics of the matched samples. **Supporting Table S3:** Baseline CCAPS‐34 scores of the matched samples. **Supporting Table S4:** Diagnostic indices for the matching procedure.

## Data Availability

Data and study materials are not publicly available but can be obtained from the first author upon reasonable request.
