# Supplementary material for: Teletherapy Post‐COVID‐19: Comparisons With In‐Person Client Characteristics and Service Utilization in Routine Practice
Source: J Clin Psychol. 2025 Aug 26;82(1):31–41. doi: 10.1002/jclp.70039 (PMC12688285; doi:10.1002/jclp.70039)
Supplement: Supplementary file 1 — Supporting Table S1: Demographic characteristics of the matched samples. Supporting Table S2: Clinical characteristics of the matched samples. Supporting Table S3: Baseline CCAPS‐34 scores of the matched samples. Supporting Table S4: Diagnostic indices for the matching procedure. [file JCLP-82-31-s001.docx]

**Supplementary Material**

**Table 1**

*Demographic characteristics of the matched samples*

| Variable | Full matched sample %  (*N* = 3,094) | In-person % (*n* = 1,547) | Telehealth %  (*n* = 1,547) | Odds ratio | *p* |
| --- | --- | --- | --- | --- | --- |
| Gender |  |  |  |  |  |
| Cisgender Man | 26.3 | 30.5 | 22.0 | 0.64 | <.001* |
| Cisgender Woman | 63.6 | 60.6 | 66.6 | 1.30 | <.001* |
| Non-Binary | 5.5 | 4.7 | 6.3 | 1.36 | .058 |
| Transgender Man | 1.4 | 1.0 | 1.7 | 1.69 | .123 |
| Transgender Woman | 0.7 | 0.5 | 0.9 | 1.99 | .187 |
| Self-Identify | 2.6 | 2.7 | 2.4 | 0.88 | .648 |
| Race/Ethnicity |  |  |  |  |  |
| African-American/Black | 10.0 | 9.2 | 10.7 | 1.18 | .187 |
| American Indian or Alaska Native | 0.4 | 0.3 | 0.5 | 1.58 | .580 |
| Asian American/Asian | 9.0 | 9.8 | 8.2 | 0.83 | .148 |
| Hispanic/Latinx | 9.4 | 9.0 | 9.8 | 1.10 | .459 |
| Native Hawaiian or Pacific Islander | 0.2 | 0.3 | 0.1 | 0.28 | .374 |
| Multi-Racial | 6.3 | 5.7 | 7.0 | 1.24 | .160 |
| White | 63.4 | 64.4 | 62.4 | 0.92 | .262 |
| Self-Identify | 1.3 | 1.4 | 1.3 | 0.95 | >.999 |
| Sexual Orientation |  |  |  |  |  |
| Asexual | 3.1 | 3.4 | 2.8 | 0.82 | .404 |
| Bisexual | 24.0 | 23.9 | 24.1 | 1.01 | .932 |
| Gay | 3.2 | 3.0 | 3.4 | 1.11 | .683 |
| Heterosexual | 49.4 | 50.0 | 48.7 | 0.95 | .517 |
| Lesbian | 3.9 | 3.9 | 3.9 | 1.00 | >.999 |
| Pansexual | 5.6 | 5.4 | 5.8 | 1.06 | .754 |
| Queer | 4.8 | 4.7 | 4.9 | 1.06 | .800 |
| Questioning | 4.6 | 4.2 | 5.0 | 1.19 | .344 |
| Self-Identify | 1.5 | 1.6 | 1.5 | 0.96 | >.999 |
| Living Situation |  |  |  |  |  |
| Alone | 16.1 | 16.2 | 16.0 | 0.99 | .960 |
| Significant Other | 12.0 | 11.8 | 12.1 | 1.02 | .868 |
| Roommates | 59.0 | 59.1 | 58.8 | 0.99 | .912 |
| Children | 1.6 | 1.6 | 1.7 | 1.04 | >.999 |
| Parents | 9.0 | 8.8 | 9.2 | 1.06 | .706 |
| Other Family | 4.3 | 4.3 | 4.3 | 1.00 | >.999 |
| Other | 1.7 | 1.9 | 1.6 | 0.86 | .680 |
| Financial Stress (*M*[*SD*]) | 2.7 | 2.8 | 2.7 | 0.09 | .016 |
| Age (*M*[*SD*]) | 21.8 | 21.5 | 22.0 | 0.11 | .002 |

*Note*. *P* values are for Fisher’s exact test.

* *p* value is significant after applying the Holm-Bonferroni correction.

**Table 2**

*Clinical characteristics of the matched samples*

| Variable | Full matched sample %  (*N* = 3,094) | In-person % (*n* = 1,547) | Telehealth %  (*n* = 1,547) | Odds ratio | *p* |
| --- | --- | --- | --- | --- | --- |
| Treatment History |  |  |  |  |  |
| Prior Psychotherapy | 74.9 | 75.0 | 74.7 | 0.98 | .868 |
| Prior Psychiatric Medication | 55.0 | 55.7 | 54.4 | 0.95 | .515 |
| Prior Psychiatric Hospitalization | 17.3 | 17.4 | 17.3 | 0.99 | .962 |
| Risk to Self and Others |  |  |  |  |  |
| Suicidal Ideation (lifetime) | 99.7 | 99.8 | 99.6 | 0.51 | .507 |
| Suicidal Ideation (past 2 weeks) | 9.1 | 9.4 | 8.9 | 0.95 | .708 |
| Homicidal Ideation (lifetime) | 10.4 | 11.1 | 9.8 | 0.87 | .238 |

*Note*. *P* values are for Fisher’s exact test.

**Table 3**

*Baseline CCAPS-34 scores of the matched samples*

| CCAPS-34 scale | Full matched sample % (*N* = 3,094) | In-person % (*n* = 1,547) | Telehealth %  (*n* = 1,547) | *t* | *p* | *d* |
| --- | --- | --- | --- | --- | --- | --- |
| Depression | 2.09 | 2.10 | 2.08 | 0.50 | .616 | 0.02 |
| Generalized Anxiety | 2.33 | 2.31 | 2.35 | 0.97 | .330 | 0.04 |
| Social Anxiety | 2.37 | 2.38 | 2.36 | 0.62 | .535 | 0.02 |
| Academic Distress | 2.23 | 2.25 | 2.21 | 1.18 | .240 | 0.04 |
| Eating Concerns | 1.31 | 1.24 | 1.37 | 2.41 | .016 | 0.09 |
| Frustration/Anger | 0.96 | 0.97 | 0.95 | 0.66 | .512 | 0.02 |
| Alcohol Use | 0.50 | 0.48 | 0.51 | 1.06 | .288 | 0.04 |
| Distress Index | 2.12 | 2.12 | 2.11 | 0.37 | .708 | 0.01 |

*Note*. Inferential statistics for Eating Concerns and Alcohol Use are based on log-transformed versions of these variables.

**Table 4**

*Diagnostic indices for the matching procedure*

| Matching variable | SMD | Maximum eCDF |
| --- | --- | --- |
| Prior psychotherapy | 0.007 | 0.003 |
| Prior psychiatric medication | 0.024 | 0.012 |
| Prior psychiatric hospitalization | 0.003 | 0.001 |
| Recent suicidal ideation | 0.015 | 0.004 |
| Gender | 0.034 | 0.060 |
| Lesbian | 0.000 | 0.000 |
| Gay | 0.017 | 0.003 |
| Bisexual | 0.004 | 0.001 |
| Questioning | 0.035 | 0.007 |
| Asexual | 0.035 | 0.005 |
| Pansexual | 0.013 | 0.003 |
| Queer | 0.012 | 0.002 |
| Heterosexual | 0.024 | 0.012 |
| Self-identify sexual orientation | 0.005 | 0.000 |
| Race/ethnicity | 0.034 | 0.020 |
| Living alone | 0.003 | 0.001 |
| Living with spouse/partner/significant other | 0.007 | 0.002 |
| Living with roommates | 0.005 | 0.002 |
| Living with children | 0.005 | 0.000 |
| Living with parent(s)/guardian(s) | 0.015 | 0.004 |
| Living with other family | 0.000 | 0.000 |
| Living with other specified | 0.020 | 0.002 |
| Baseline Distress Index | 0.013 | 0.030 |

*Note*. SMD: Standardized mean difference. eCDF: empirical cumulative distribution function. The SMD for the joint distribution of all covariates (distance) was 0.003, and the Maximum eCDF was 0.007.
